# *BCR::ABL1*-negative myeloproliferative neoplasms in the era of next-generation sequencing

**DOI:** 10.3389/fgene.2023.1241912

**Published:** 2023-09-08

**Authors:** Aleksandra Mroczkowska-Bękarciak, Tomasz Wróbel

**Affiliations:** Department of Hematology, Blood Neoplasms and Bone Marrow Transplantation, Wroclaw Medical University, Wrocław, Poland

**Keywords:** hematooncology, myeloproliferative neoplasms, gene mutations, next-generation sequencing, polycythemia vera, essential thrombocythemia, myelofibrosis

## Abstract

The classical *BCR::ABL1*-negative myeloproliferative neoplasms such as polycythemia vera (PV), essential thrombocythemia (ET), and myelofibrosis (MF) are clonal diseases with the presence of characteristic “driver mutations” in one of the genes: JAK2, CALR, or MPL. The search for mutations in these three genes is required for the diagnosis of MPNs. Nevertheless, the progress that has been made in the field of molecular genetics has opened a new era in medicine. The search for additional mutations in MPNs is helpful in assessing the risk stratification, disease progression, transformation to acute myeloid leukemia (AML), or choosing the right treatment. In some cases, advanced technologies are needed to find a clonal marker of the disease and establish a diagnosis. This review focuses on how the use of new technologies like next-generation sequencing (NGS) helps in the diagnosis of *BCR::ABL1*-negative myeloproliferative neoplasms.

## 1 Introduction

The classical *BCR::ABL1*-negative myeloproliferative neoplasms (MPNs) are hematopoietic stem cell disorders with the clonal proliferation of one or more types of cells of the myeloid lineage. MPNs are associated with an increased risk of thrombosis and a tendency to transform into acute myeloid leukemia. According to the fifth edition of the World Health Organization (WHO) criteria, MPNs include chronic myeloid leukemia (CML), which is defined by the presence of the Philadelphia chromosome and *BCR::ABL1* fusion gene, polycythemia vera (PV), essential thrombocythemia (ET), primary myelofibrosis (PMF), chronic neutrophilic leukemia (CNL), chronic eosinophilic leukemia (CEL), juvenile myelomonocytic leukemia, and myeloproliferative neoplasms, not otherwise specified. PV, ET, and PMF are referred to as classical *BCR::ABL1*-negative MPNs. CNL, CEL, juvenile myelomonocytic leukemia, and myeloproliferative neoplasms, not otherwise specified, are less common and called non-classical or atypical MPNs. The current criteria for the diagnosis of myeloproliferative neoplasms are provided by the fifth edition of the World Health Organization Classification of Haematolymphoid Tumours: Myeloid and Histiocytic/Dendritic Neoplasms, and the International Consensus Classification (ICC) of Myeloid Neoplasms and Acute Leukemias. The diagnosis still depends on the clinical picture, laboratory tests, evaluation of bone marrow biopsy, and molecular tests for the presence of mutations in the *JAK2*, *CALR*, or *MPL* genes. These mutations, called “driver mutations,” are crucial for diagnosis and have prognostic and therapeutic significance (Barbui et al., 2018; Arber et al., 2022; Khoury et al., 2022).

**FIGURE 1:**
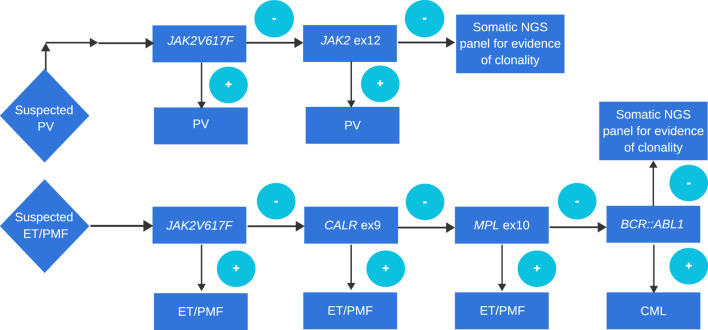
Approach to genetic diagnosis of PV, ET, and PMF. A cascade approach is routinely used in many laboratories. Although more and more often if the probability of MPN is high in order to simultaneously search for driver mutation, other evidence of clonality, and search for mutations in genes of prognostic relevance, a panel testing approach is performed.

The diagnostic criteria for PV have been slightly refined. One of the major criteria, namely, increased red cell mass, has been removed as a diagnostic criterion according to the WHO classification. However, the ICC classification still retains this criterion (Table 1). The diagnostic criteria of ET have not changed (Table 2). In the diagnosis of primary myelofibrosis, it is necessary to not only differentiate prefibrotic PMF from ET and PV but also from fibrotic PMF. Serial monitoring of the spleen size and bone marrow fibrosis using repeatable and defined criteria is still important, especially for patients using JAK1/2 inhibitors (Table 3) (Levine et al., 2005; Khoury et al., 2022). The presence of *JAK2*V617F, *JAK2* exon 12, *MPL*, and *CALR* mutations is included in the molecular diagnostic and prognostic algorithms for MPNs. All these mutations lead to the constitutive activation of the JAK-STAT signaling pathway. Furthermore, these genetic mutations are often considered mutually exclusive. The absence of driver mutations does not exclude diagnosis; these patients are referred to as triple-negative (TN) patients. Nevertheless, driver mutations are not the only mutations identified in patients with MPNs. The use of advanced techniques of molecular genetics, such as NGS, plays a very important role in hematooncology diagnosis. The development of high-throughput NGS methods diversified the mutational landscape and improved our understanding of pathophysiology. These molecular details also made it possible to improve the diagnosis, assign a better prognosis score, and track the effectiveness of treatments. Comprehensive gene sequencing of blood cancer patients is becoming more widely available. We wanted to share a graphical flowchart of stepwise molecular testing ending with NGS (Figure 1). Somatic additional mutations were revealed to affect a number of genes that were often altered throughout myeloid malignancies and MPNs. More than 50% of patients with MPNs have additional mutations in these myeloid cancer genes, which occur more frequently as people get older and most frequently in patients with PMF. The mutational profile in conjunction with cytogenetic, histopathological, hematological, and clinical variables plays an increasingly important role in the risk stratification of patients in terms of thrombosis, overall survival, and the rate of transformation to secondary myelofibrosis or secondary leukemia.

**TABLE 1:** Diagnostic criteria for polycythemia vera (PV) by Khoury et al. (2022) and Arber et al. (2022).

Polycythemia vera	Post-PV MF
Major criteria	Required criteria
1. Elevated hemoglobin concentration	1. Previously established diagnosis of PV
HGB>16.5 g/dL (men), >16 g/dL (women), or elevated hematocrit HCT> 49% (men)	2. Bone marrow fibrosis of grade 2 or 3
>48% (women) or increased red blood cell mass: >25% above mean normal predicted value	
2. Presence of *JAK2V617F* or *JAK2* exon 12 mutation	
3. Bone marrow biopsy showing age-adjusted hypercellularity with proliferation in three hematopoietic lineages including evident erythroid, granulocytic, and megakaryocytic lineages with mature megakaryocytes without of various size	
Minor criterion	Additional criteria
1. Subnormal serum erythropoietin (EPO) level	1. Anemia (i.e., below the reference range given age, sex, and altitude considerations) or sustained loss of requirement of either phlebotomy (in the absence of cytoreductive therapy) or cytoreductive treatment for erythrocytosis
	2. Leukoerythroblastosis
	3. Spleen enlargement greater than 5 cm from baseline or development of newly palpated splenomegaly
	4. Evolvement of any two (or all three) of the following constitutional symptoms: >10% weight loss in 6 months, night sweats, and unexplained fever (>37.5°C)
Diagnosis of PV requires either all three major criteria or the first two major criteria and the minor criterion	All required criteria plus at least two additional criteria are necessary to determine the post-PV MF

**TABLE 2:** Diagnostic criteria for essential thrombocythemia (ET) by Khoury et al. (2022) and Arber et al. (2022).

Essential thrombocythemia	Post-ET MF
Major criteria	Required criteria
1. Platelet count ≥450 × 10^9^/L	1. Previously established diagnosis of ET
2. Bone marrow biopsy showing proliferation mostly of the megakaryocytic lineage, with increased numbers of enlarged, mature megakaryocytes with hyperlobulated nuclei; no significant increase or left shift in neutrophil granulopoiesis or erythropoiesis and a very rarely minor (grade 1) increase in reticulin fibers	2. Bone marrow fibrosis of grade 2 or 3
3. Not meeting WHO criteria for *BCR::ABL1*-positive CML, PV, PMF, or other myeloid neoplasms	
4. Presence of *JAK2*, *CALR*, or *MPL* mutations	
Minor criterion	Additional criteria
1. Presence of a clonal marker or the absence of evidence of reactive thrombocytosis	1. Anemia (i.e., below the reference range, given age, sex, and altitude considerations) and a >2 g/dL decrease from the baseline hemoglobin concentration
	2. Leukoerythroblastosis
	3. Increase in palpable splenomegaly of >5 cm from baseline or the development of a newly palpable splenomegaly
	4. Elevated LDH level above the reference range
	5. Development of any two (or all three) of the following constitutional symptoms: >10% weight loss in 6 months, night sweats, and unexplained fever (>37.5°C)
Diagnosis of ET requires all four major criteria or the first three major criteria plus a minor criterion	All required criteria plus at least two additional criteria are necessary to determine the post-ET MF.

**TABLE 3:** Diagnostic criteria for primary myelofibrosis (PMF) by Khoury et al. (2022) and Arber et al. (2022).

Myelofibrosis, early/pre-fibrotic stage	Myelofibrosis, overt-fibrotic stage
Major criteria	
1. Bone marrow biopsy showing megakaryocytic proliferation and atypia, without reticulin fibrosis > grade I, goes along with increased age adjusted BM cellularity, granulocytic proliferation, and often decreased erythropoiesis	1. Bone marrow biopsy showing megakaryocytic proliferation and atypia, accompanied by reticulin and/or collagen fibrosis grades 2 or 3
2. *JAK2*, *CALR*, or *MPL* mutations or the presence of another clonal marker or the absence of minor reactive bone marrow reticulin fibrosis	2. *JAK2*, *CALR*, or *MPL* mutations or the presence of another clonal marker or the absence of reactive myelofibrosis
3. Not meeting WHO criteria for *BCR::ABL1*-positive CML, PV, ET, myelodysplastic syndromes, or other myeloid neoplasms	3. Not meeting WHO criteria for ET, PV, *BCR*::*ABL1*-positive CML, myelodysplastic syndrome, or other myeloid neoplasms
Minor criteria	
1. Anemia not attributed to a comorbid condition	1. Anemia not attributed to a comorbid condition
2. Leukocytosis >11 × 10^9^/L	2. Leukocytosis ≥11 × 10^9^/L
3. Palpable splenomegaly	3. Palpable splenomegaly
4. LDH level above the reference range	4. LDH level above the reference range
	5. Leukoerythroblastosis
Diagnosis of PrePMF requires all three major criteria and at least one minor criterion	Diagnosis of overt-PMF requires all three major criteria and at least one minor criterion

## 2 Classical *BCR::ABL1*-negative MPNs

### 2.1 JAK2

JAK2 is a non-receptor tyrosine kinase that is involved in hematopoietic cytokine signaling through erythropoietin, thrombopoietin, and granulocyte colony-stimulating growth factor receptors. The normal function of this signaling pathway ensures proper regulation of cell proliferation, survival, differentiation, and immune response. *JAK2* gene mutations lead to constitutive activation of the STAT signaling pathway and uncontrolled cell proliferation. The most common mutation in myeloproliferative neoplasms, which was discovered in 2005, is the G to T transversion in exon 14, resulting in a valine to phenylalanine substitution at codon 617 (Baxter et al., 2005; James et al., 2005; Kralovics et al., 2005; Levine et al., 2005; Guglielmelli et al., 2017; Loscocco et al., 2021a). The *JAK2V617F* mutation is detected in approximately 98% of patients with PV and 50%–60% of patients with ET and PMF. Almost 2%–3% of patients with PV who are *JAKV617F*-negative may harbor rare insertions or deletions in exon 12 of the *JAK2* gene. The *JAK2V617F* mutation is usually detected by allele-specific PCR (Baxter et al., 2005; Scott et al., 2007; Scott, 2011; Tefferi et al., 2016a). Quantitative PCR methods such as RQ-PCR, ddPCR (droplet digital PCR), and NGS (next-generation sequencing) can also be used to support the diagnosis of PV, ET, or MF to separate triple-negative cases and monitor MRD (minimal residual disease) (Haslam and Langabeer, 2016; Link-Lenczowska et al., 2018; Arber et al., 2022). Peripheral blood counts, in patients with this mutation, show erythrocytosis, thrombocytosis, and/or leukocytosis. The *JAK2* mutation status is widely known as the thrombosis risk factor and disease risk progression in patients with MPNs (Zhang et al., 2020). PMF patients with *JAK2* mutations have been associated with older age, higher hemoglobin levels, lower platelet counts, and a higher level of leukocytes (Vannucchi et al., 2013). Mutations in *JAK2V617F* are one of the four risk factors in the International Prognostic Score of thrombosis for ET (IPSET-thrombosis) (Barbui et al., 2012). Additionally, *JAK2V617F* allele burden can provide important prognostic information, which has been associated with hematologic parameters and clinical outcomes. Patients with PV and higher *JAK2V617F* allele burdens have been associated with a higher hemoglobin level, increased age, pruritus, presence of splenomegaly, a higher risk of thrombotic events, and disease progression (Tefferi et al., 2016a; Sazawal et al., 2019; Guglielmelli et al., 2021a; Lee et al., 2021; Regimbeau et al., 2022). In ET patients, the *JAK2V1617* allele burden level has been correlated with older age and a higher hemoglobin level, which is associated with risk factors for thrombotic events (Lee et al., 2021). High *JAK2V617F* allele burden is associated with disease progression in PMF cases. Studies have shown that allele burden may vary across different MPN phenotypes. The lowest allele burden has been associated with ET cases, and a higher allele burden has been observed in PV and PMF cases (Park et al., 2013; Vannucchi et al., 2013; Barosi et al., 2017; Lee et al., 2021; Loomila et al., 2022). The quantification of *JAK2V617F* allele burden is a helpful marker of treatment response and a measure of MRD after stem cell transplantation. However, there is a certain degree of overlap, and allelic burden cannot be considered pathognomonic of disease (Rumi et al., 2020; Sørensen et al., 2020; Stegelmann et al., 2023).

### 2.2 *CALR*

*CALR* is a highly conserved protein that is localized in the endoplasmic reticulum. It is involved in the quality control of N-glycosylated proteins and the modulation of calcium ion homeostasis. Mutations in this gene are the second most common genetic abnormality in myeloproliferative neoplasms after *JAK2V617F* gene mutations. This type of mutation is not found in patients with PV. *CALR* mutations are detected in about 20%–25% ET cases and 35% PMF (Constantinescu and Pecquet, 2020). The first studies about calreticulin date back to 2013 (Klampfl et al., 2013; Nangalia et al., 2013). The two most prevalent mutations, detected in exon 9 and corresponding to 52-bp deletion (p.Leu367ThrfsTer46), are recognized as type 1, and that with a 5-bp insertion (p.Lys385AsnfsTer47) is called type 2. Other insertions and deletions occurring at a lower frequency are reported as type like-1 or type like-2 mutations. Although type 1 and type 2 mutations account for 80% of all mutations, type 1 *CALR* mutation is much more common than type 2. Among ET patients, type 1 occurs in approximately 55% of cases and type 2 occurs in approximately 35% of cases; however, in PMF, type 1 definitely predominates and occurs in approximately 75% of cases, whereas type 2 occurs in only 15% of cases (Cabagnols et al., 2015; Vainchenker and Kralovics, 2017; How et al., 2019). Studies have shown that *CALR* mutations can activate the thrombopoietin receptor MPL and afterward the JAK-STAT signaling pathway (Chachoua et al., 2016; Nivarthi et al., 2016). Clinical characteristics and outcomes are different between patients who have *CALR* mutations and patients with *JAK2* mutation, even though they both activate the same JAK/STAT signaling pathway. *CALR* mutant patients have lower leukocyte counts, lower hemoglobin levels, higher platelet counts, lower thrombosis risks, and better survival than *JAK2* mutant patients (Klampfl et al., 2013; Rotunno et al., 2014). The presence of mutations in the *CALR* gene in ET patients is associated with a lower risk of thrombosis (Tefferi et al., 2014a; Rotunno et al., 2014; Pei et al., 2016; Guglielmelli et al., 2021b; Zulkeflee et al., 2021). It seems that both *CALR* and *JAK2* mutations have a similar risk of transformation to post-ET myelofibrosis (Tefferi et al., 2014b; Rotunno et al., 2014; Rumi et al., 2014). However, some studies have shown higher risks of fibrotic progression in *CALR*-mutated than *JAK2*-mutated patients with ET (Al Assaf et al., 2015; Grinfeld et al., 2018). More detailed analyses have shown that type 1 of the *CALR* mutation is associated with an increased risk of transformation to MF (Pietra et al., 2016; Loscocco et al., 2021b; Weir et al., 2023). Little information is accessible about the allele burden of *CALR* mutations. A recent paper about correlations of the *CALR* mutation variant allele frequency (VAF) in patients with myelofibrosis showed an association with a higher VAF of *CARL*, a lower hemoglobin level and platelet count, higher peripheral blood CD34^+^ cell counts, the need for cytoreduction therapy, and shorter leukocytosis-free survival. In addition, the authors emphasize that high-molecular risk mutations (HMR) were more frequent in *CALR* patients with high VAF (Guglielmelli et al., 2023). Other studies have focused on various phenotypic impacts of the *CALR* allele burden, according to the type of *CALR* mutant. They have found that in patients with type 1-like *CALR* mutation, *CALR* mutant burden was negatively correlated with the hemoglobin level and platelet count, whereas *CALR* mutant burden was positively correlated with absolute neutrophil count and platelet count with type 2-like *CALR*-mutated patients. Nevertheless, the study was conducted on a small number of patients (Kim et al., 2022). There are several methods for detecting the *CALR* mutation. The most common methods are fragment analysis PCR and high-resolution melting-curve (HRM) analysis. Sanger sequencing can be useful in confirming the type of mutation; however, this technique has limited sensitivity. Next-generation sequencing (NGS) has the lowest limit of detection (LOD), but it is quite expensive for routine diagnostics (Jones et al., 2015; Giannopoulos et al., 2019).

### 2.3 MPL

The myeloproliferative leukemia virus oncogene (MPL) encodes a receptor for thrombopoietin that regulates megakaryopoiesis and platelet production, and is also crucial in the self-renewal of hematopoietic stem cells. Mutations in the *MPL* gene activate JAK2 and the thrombopoietin pathway. The first studies on the *MPL* mutant in ET and MF patients were published in 2006. MPL mutations are detected in 5%–10% of all myelofibrosis patients and in 1%–4% of patients with essential thrombocythemia. All mutations are located in exon 10. The most common mutations are substitutions at the Trp515 position, W515L, and W515K. Other mutations in that position are very rare (Pikman et al., 2006; Defour et al., 2016; Guglielmelli and Calabresi, 2021). Patients with ET or MF and *MPL* mutations have lower hemoglobin and hematocrit values in relation to higher EPO levels. The presence of this mutation in ET patients is associated with a higher risk of transformation to MF or AML (Asp et al., 2016). Mutations in the *MPL* gene can be detected in several ways, such as an allele-specific PCR-based strategy, Sanger sequencing, real-time qPCR assay, ARMS-PCR, HRM curve, and NGS. In recent years, most of the methods for detecting mutations in the *MPL* gene have been improved. Most of these techniques are sensitive, efficient, and cost-effective (Zhuge et al., 2010; Furtado et al., 2013; Arunachalam et al., 2018; Ullah et al., 2022).

### 2.4 Triple-negative MPN

*JAK2*, *CALR*, and *MPL* can be seen in about 90% of MPN cases; nevertheless, in 2% of PV, 15% of ET patients, and approximately 8%–10% of MF cases, driver mutations are not detected; such patients are termed TN patients. Some patients may have rare mutations in the *JAK2*, *MPL*, and *CALR* genes that are not detected by routine diagnostics. In other cases, the mutation may have been at a very low mutant allele burden and can be detected only by applying a more sensitive methodology. Implementation of more advanced techniques of molecular biology, like next-generation sequencing, allowed to improve the understanding of triple-negative patients and revealed the presence of non-driver mutations. NGS helps in finding a clonal marker to confirm the diagnosis. Triple-negative PMF patients were associated with poor survival and increased leukemic transformation. These patients tend to have lower Hb levels, platelet and leukocyte counts, and a higher IPSS (International Prognostic Scoring System) risk. TN-ET patients are associated with a low vascular event rate and a lower risk of leukemic transformation compared to TN-MF patients (Tefferi et al., 2018a; Szuber and Tefferi, 2018; Cattaneo et al., 2021; Michail et al., 2021).

## 3 Next-generation sequencing in MPNs

The amazing progress of genetics over the last decade has changed the oncological diagnosis. Thanks to the development of advanced techniques in molecular biology, we have learned the pathomechanisms of numerous cancers. Indeed, knowledge about genetic changes in cancer has allowed the creation of diagnostic and prognostic models. NGS is a technique based on reading the genetic sequence nucleotide by nucleotide. This method allows the transition from the analysis of small gene fragments by traditional methods to the use of techniques capable of obtaining the complete sequence of many genes or even the entire genome. There are many NGS platforms with different sequencing chemistry and signal detection methods. The use of advanced techniques of molecular genetics such as NGS plays a very important role in hematooncology diagnostics. Despite the fact that the routine clinical usage of targeted or complete gene sequencing of hematological cancer patients is becoming more and more accessible, there are still numerous unresolved issues. The rapid expansion of the number of genes under consideration, the prevalence of variants of uncertain significance (VUSs) in less well-studied genes, the need to distinguish between germline and somatic mutations, and the lack of established guidelines regarding which patients and which genes should be tested are all challenges to clinical testing for these neoplasms. The most important aspect is data analysis, where bioinformatic tools are needed for mapping the reads to the human reference genome. Clarifying the somatic vs. germline origin of the mutations is one of the biggest problems in using and interpreting NGS data. Filtering NGS results would require a reliable method to distinguish between somatic and germline mutations. Variant categorization and annotation, including classifying variants into distinct “tiers” based on pathogenicity or clinical importance, may differ between laboratories in addition to interlaboratory variations in VAFs and the assignment of somatic status to variants. In general, it is recommended that interpretations follow specialist guidelines, such as those provided by the College of American Pathologists, the American Society for Clinical Oncology, and the Association for Molecular Pathology (Li et al., 2017). Despite all these disadvantages, genome profiling becomes the basis for prognosis prediction that is specifically tailored to each patient, but it is important to realize that the practical application of the latest scientific developments is still in progress. Over the past decade, tremendous progress has been made in understanding the pathogenesis of *BCR::ABL1*-negative myeloproliferative neoplasms. The dynamic development of genetics and the use of NGS in diagnostics have opened a new era in medicine. Additional mutations occur in more than 50% of patients with PV/ET and 80% of PMF cases. However, these mutations are not unique to MPN but are characteristic of all hematological malignancies. Most often these additional mutations occur in genes related to epigenetic regulation (*TET2*, *DNMT3A*, *IDH1/2*, *ASXL1*, and *EZH2*), mRNA splicing (*SF3B1*, *SRSF2*, *U2AF1*, and *ZRSR2*), signaling pathways (*NRAS*, *KRAS*, *CBL*, *NF1*, *SH2B3*, and *PTPN11*), and transcription factors (*RUNX1* and *TP53*) (Tefferi et al., 2016a; Tefferi et al., 2016b; Luque Paz et al., 2023). The approximate frequency of additional MPN somatic mutations is shown in Table 4. The main target of myeloproliferative neoplasm therapy is based on the treatment of related symptoms, avoiding thrombosis and bleeding, improving the quality of life, and minimizing the risk of transformation to AML and post-PV/ET myelofibrosis. Until recently, information obtained from studies using advanced molecular techniques, such as NGS, contributed to improving the diagnosis and identification of new molecular biomarkers, establishing more accurate risk assessments, and selecting more individual therapeutic interventions. Testing for additional mutations has an impact on the therapeutic decision-making process. Data obtained from the NGS enable the creation of prognostic scoring systems based on genetic changes. An example is the GIPSS scale used in the assessment of prognostic risks in PMF. However, Grinfeld et al. created multi-stage prognostic models for specific individuals based on the genomic classification for all myeloproliferative neoplasms. This model identifies patients with chronic-phase MPN who are at a high risk of disease progression. Such patients might be taken into account for clinical trials of novel therapeutics. This model also identifies the majority of patients who initially appeared to have a good prognosis. Among these patients, a conservative therapeutic approach focusing on cytoreduction and vascular risk reduction will be sufficient. NGS opens up the possibility of using targeted therapies due to the presence of a mutation in a specific gene (Grinfeld et al., 2018; Loscocco et al., 2020; Luque Paz et al., 2023).

**TABLE 4:** Approximate frequency of additional MPN somatic mutations by Tefferi et al. (2016a), Tefferi et al. (2016b), Senín et al. (2018), and Vannucchi et al. (2013).

Gene	PV	ET	PMF	Prognostic impact
Epigenetic regulation				
*TET2*	10%–22%	3%–10%	10%–20%	No defined prognostic effect
*ASXL1*	2%–7%	5%–10%	15%–35%	Inferior survival, risk of fibrotic, and leukemic transformation
*DNMT3A*	5%–10%	1%–5%	8%–12%	Interior overall survival
*EZH2*	1%–2%	1%–2%	7%–10%	Inferior survival, risk of fibrotic, and leukemic transformation
*IDH1*	1%–2%	1%–2%	5%–6%	Interior overall survival
*IDH2*	1%–2%	1%–2%	5%–6%	Interior overall survival
mRNA splicing				
*SRSF2*	<2%	<2%	6%–14%	Increased risk of leukemic transformation and inferior overall survival
*U2AF1*	<2%	<2%	7%–10%	Reduced overall survival in MF and associated with disease progression
*SF3B1*	2%–3%	2%–5%	5%–7%	Associated with ring sideroblasts and an increased risk of fibrotic transformation
Signaling pathway				
*SH2B3*	2%–9%	1%–3%	2%–4%	Role in leukemic transformation
*CBL*	<2%	<2%	4%	Reduced overall survival in MF and resistance to ruxolitinib
*NRAS/KRAS*	<2%	<2%	2%–4%	Reduced overall survival in MF and resistance to JAKi
Transcriptional factor				
*RUNX1*	<2%	<2%	2%–3%	Increased risk of leukemic transformation and inferior overall survival
*TP53*	<2%	<2%	4%–5%	Very unfavorable. Associated with disease progression and reduced overall survival in all MPNs

### 3.1 Essential thrombocythemia/polycythemia vera

#### 3.1.1 Prognosis of somatic mutations

In recent years, research has focused on the significance of additional mutations in MPN patients. Using a targeted sequencing panel of 27 genes, Tefferi et al. observed that the most common mutations among ET patients were *TET2* and *ASXL1* genes. In addition, mutations in *TET2* and *SF3B1* were associated with an older age, *SF3B1* with higher platelet counts, and *ASXL1* with palpable splenomegaly. Mutations in the *IDH2*, *EZH2*, and *SH2B3* genes were crucial as risk factors for survival in ET patients. Leukemia-free survival was associated with mutations in *TP53*, *EZH2*, *SRSF2*, and *IDH2*, while myelofibrosis-free survival was associated with *SF3B1* and *U2AF1* mutations. This study identified *SH2B3*, *IDH2*, *U2AF1*, *SF3B1*, *EZH2*, and *TP53* variants as being “adverse” in patients with ET. The most common mutations among PV patients were also *TET2* and *ASXL1* genes. A phenotypic correlation in PV was observed only between *SH2B3* and palpable splenomegaly. In PV patients, mutations in *SRSF2*, *ASXL1*, and *IDH2* were associated with inferior survival. Significant association was also noted between *SRSF2*, *IDH2*, and *RUNX1* for leukemia-free survival, and SRSF2 and *RUNX1* for myelofibrosis-free survival. Considering overall, leukemia-free or myelofibrosis-free survival, *ASXL1*, *SRSF2*, and *IDH1* mutations were related as “adverse.” Through the use of next-generation sequencing, this work has defined mutations that were associated with poorer overall survival (Tefferi et al., 2016a). Another study has shown that there is an association between additional mutations and clinical outcomes in patients with PV and ET. Using a panel of 51 genes, Senin et al. analyzed mutational profiles in 100 patients. The most common mutations occurred in the following genes: *TET2*, *DNMT3A*, *TP53*, and *ASXL1*. Shorter survival was associated with mutations in *DNMT3A*, *SRSF2*, *SF3B1*, *IDH1/2*, and *RUNX1*. The presence of additional mutations was associated with patients who transformed into AML or MF. These patients have developed new mutations more frequently during the time of transformation, which suggests genetic instability. Patients with mutations in *SF3B1* and *IDH1/2* had a higher risk of transformation to MF. Mutations in the *ASXL1*, *TP53*, *IDH1/2*, *SRSF2*, and *RUNX1* genes have been associated with the risk of transformation to AML. They also noticed that the VAF for some mutations of the observed patients increased compared to the VAF from the time of diagnosis (Senín et al., 2018). Similarly, Luque Paz et al. observed that the most frequent mutations in patients with PV and ET were also *TET2*, *ASXL1*, *IDH1/2*, and *DNMT3A.* They observed that patients with additional mutations had evidence of progression at 3 years. In these kinds of patients, they noticed mutations in the *ASXL1*, *IDH1/2*, and *SRSF2* genes. Studies also revealed that allele burden increased between diagnosis and follow-up. Furthermore, next-generation sequencing helped establish that some of the additional mutations observed during the follow-up also existed at the time of diagnosis in VAF <2% (Luque Paz et al., 2017). In another study, Laque Paz et al. investigated the time of leukemic transformation in PV and ET patients using a panel of 52 genes. ET patients with *CALR* mutation showed late transformation. The most recurrent mutated genes were *TP53*, *TET2*, *RUNX1*, *ASXL1*, and *EZH2*. Mutations in the following genes were associated with late transformation: *TP53*, *BCORL1*, and *NRAS*. *U2AF1*, *IDH1/2*, *EZH2*, and *DNMT3* were linked to an earlier time of transformation (Luque Paz et al., 2020). Many other publications confirm that additional mutations in PV and ET are frequent and are associated with inferior overall survival and increased risks of transformation to MF or AML (Lasho et al., 2018; Pasca et al., 2022; Morishita et al., 2023).

#### 3.1.2 Treatment

A few studies have shown associations between treatment response and high-risk mutations. Intolerance to hydroxyurea (HU) occurs in 20%–30% of PV patients, and this has been correlated with an increased risk of thrombosis, disease progression, and shorter survival. The possibility of evolving resistance to HU treatment has been estimated at 64% in patients with the *TP53* mutation and at 49% in patients with spliceosome or chromatin gene mutations (Alvarez-Larrán et al., 2021). Some of the additional mutations were correlated with an increased risk of developing cytopenias during HU treatment (Senín et al., 2018). Similar observations were made in the response of ET patients with *CALR* mutations to interferon alpha. The presence of *TET2*, *IDH2*, *ASXL1*, and *TP53* mutations was associated with a poorer response to therapy (Verger et al., 2015). The presence of additional mutations in the *TET2*, *ASXL1*, *DNMT3A*, *IDH*, and *EZH2* genes in PV and ET patients was associated with failure to achieve a complete molecular response during treatment with pegylated interferon alpha (Quintás-Cardama et al., 2013). Another study has revealed that failure to achieve a complete molecular response is associated with *DNMT3A* mutations (Knudsen et al., 2022).

#### 3.1.3 History of thrombosis

Tefferi et al. observed that as for a phenotypic correlation, patients with ET and *TET2* mutations were associated with an increased risk of thrombosis, which was independent of driver mutation status and age. Nevertheless, such an association was not noticed in a cohort of Italian patients with ET (Tefferi et al., 2016a). Another study also highlighted that there may be an association between the history of thrombosis and the presence of *TET2*, *DNMT3A*, or *ASXL1* mutations. However, different studies on 587 patients did not find a significant influence on either arterial or venous events (Cerquozzi et al., 2017; Segura-Díaz et al., 2020).

### 3.2 Myelofibrosis

Among patients with myelofibrosis, additional mutations are more frequent than those in other myeloproliferative neoplasms. These are mutations in genes involving DNA methylation, chromatin modification, RNA splicing, or DNA repair.

#### 3.2.1 Diagnosis

According to the latest recommendations, in the absence of driver mutations, the detection of other mutations in genes such as *ASXL1*, *EZH2*, *IDH1*, *IDH2*, *SF3B1*, *SRSF2*, and *TET2* supports the clonal nature of the disease. This information is included as a major criterion for pre-PMF and over-fibrotic PMF stages (Arber et al., 2022). Additional mutations in myelofibrosis have been studied for several years because of the worse prognosis and higher risk of transformation to AML than PV or ET. The first publication about additional mutations in myelofibrosis appeared in 2013.

#### 3.2.2 Prognosis of somatic mutations

Vannucchi et al. investigated 879 patients. A study revealed that in the European cohort, *ASXL1*, *EZH2*, and *SRSF2* were associated with shorter survival. This has been confirmed in another Mayo Clinic group where mutations in the *ASXL1*, *SRSF2*, and *EZH2* genes were linked to poor survival. Increased risks of leukemic transformation were also associated with mutations in *ASXL1*, *SRSF2*, and *IDH1/2* genes in the European group. In the Mayo Clinic group, leukemia-free survival was associated with *IDH1* and *SRSF2* mutations. Mutations in the *ASXL1*, *SRSF2*, *EZH2*, and *IDH1/2* genes, referred to as high-risk mutations (HRM), identify patients who have a higher risk of transformation to AML or a higher risk of death (Vannucchi et al., 2013). Guglielmelli et al. searched for a codependence between the number of HMR mutations and their effects on OS and leukemia-free survival (LFS). Patients with two or more additional high-risk mutations (*ASXL1*, *EZH2*, *SRSF2*, and *IDH1/2*) had decreased OS and LFS compared to patients with only one additional mutation or those without any (Guglielmelli et al., 2014). Tefferi et al. showed that the most frequent genes in patients with MF were *ASXL1*, *TET2*, *SRSF2*, *U2AF1*, *ASXL1*, *SRSF2*, *CBL*, *KIT*, *RUNX1*, *CEBPA*, and *SH2B3*, which have been associated with poorer overall survival and leukemia-free survival. Studies have confirmed that the number of additional mutations matters; however, analyses have shown that patients with one or two adverse mutations have similar survival but inferior survival compared to patients who had no additional mutations and superior to those who had three or more (Tefferi et al., 2016b). Studies conducted using a targeted panel of 137 genes on the cohort of 259 patients with pre-PMF, overt-PMF, and PMF-AP/BP have shown that the most recurrent additional mutations were in *ASXL1*, *TET2*, *SRSF2*, *U2AF1*, and *SETBP1*. Mutations in *ASXL1* and *U2AF1* were more frequent in overt-PMF than those in pre-PMF. Mutations in *ASXL1*, *SRSF2*, *RUNX1*, *SETBP1*, *NOTCH2*, *NRAS*, and *EZH2* were presented more frequently in PMF-AP/BP than those in overt-PMF. They have also noticed that the smaller clone size of the *ASXL1* mutation had adverse risk factors in overt-PMF and PMF-AP/BP patients (Yan et al., 2022). Another study on 113 patients with overt-PMF and pre-fibrotic PMF has shown similar results. The most frequent additional mutations were observed in *ASXL1*, *TET2*, *EZH2*, *DNMT3A*, *SRSF2*, *SF3B1*, *U2AF1*, *TP53*, and *IDH1/2*. No significant differences were observed between overt-PMF and pre-fibrotic-PMF patients. Those patients with a high-risk mutation (*ASXL1*, *EZH2*, and *SRSF2*) have been associated with poorer prognosis and disease progression. In these groups of patients, leukemic transformation was detected only in patients with high-risk mutations (Morishita et al., 2021). In another study, patients with PMF were compared to patients with PV and ET. The frequencies of high-risk molecular mutations were significantly higher in patients with PMF than those in PV and ET patients. In addition, allele burdens of *TET2* and *DNMT3A* mutations were higher in patients with PV and PMF than those in ET patients. This may suggest that *TET2* and *DNMT3A* mutations may escalate due to the acquisition of HMR mutations in PMF and thus lead to a worse prognosis (Morishita et al., 2023). Laque Paz et al., as in other studies, observed that the most frequently mutated genes in myelofibrosis were *ASXL1*, *TET2*, *SRSF2*, *U2AF1*, and *EZH2*. In addition, they made very interesting observations dividing patients into four groups, namely, *TP53*-mutated patients, patients with ≥1 mutation in *EZH2; CBL*, *U2AF1*, *SRSF2*, *IDH1*, *IDH2*, *NRAS*, or *KRAS* - high-risk groups; ASXL1-only mutation; and “other” patients mainly mutated in the *NFE2*, *DNMT3A*, *TET2*, and *SF3B1* genes. The worst prognostic effects on OS, leukemic transformation, and leukemia-free survival were in the TP53 and high-risk groups compared to those in the ASXL1-only and “other patient” groups. Moreover, the TP53 mutation and high-risk groups were correlated with a higher risk of non-AML death. Interestingly, they noted that the ASXL1-only mutation group did not have an adverse prognosis on OS and only a moderate effect on leukemic transformation. They suggested that ASXL1 mutations themselves had no negative effects but appeared to confer an adverse risk factor when correlated with high-risk mutations (Luque Paz et al., 2021). Guglielmelli et al. also investigated the prognostic role of *ASXL1* but with a distinction between PMF and secondary MF (SMF). They also divided the patients into four categories: *TP53* group, high-risk group, *ASXL1* only, and “others.” Patients in the *TP53* and *ASXL1*-only groups were more frequently diagnosed with SMF. Patients with *TP53* and the high-risk group had the worst OS. Among patients with PMF, a negative prognostic effect of ASXL1-only was noted in comparison with the “others” category. In PMF, *ASXL1* mutations among patients from the high-risk group mutation were associated with shorter OS. In PMF, VAF for *ASXL1* in the *TP53* and high-risk group was greater than those for ASXL1-only. Unlike SMF, VAF was lower in those groups, which may suggest that *ASXL1* mutations are early driving events in PMF, but in SMF, these mutations might be acquired later. In SMF, the worst OS had the *TP53* group. They noted that there were no differences between the OS of the ASXL1-only group and “other” or high-risk groups. In SMF, *ASXL1* mutations in the high-risk group had no impact on OS. These recent studies have emphasized the concept that PMF and SMF are different biological entities; furthermore, they also confirmed the adverse effect of the *ASXL1* mutation on PMF not in SMF (Guglielmelli et al., 2022).

#### 3.2.3 Treatment

Notably, *RAS/CBL* mutations in PMF were associated with poor response to ruxolitinib treatment and reduced survival. The authors suggested that it is important to identify *RAS* mutations in patients with MF (Coltro et al., 2020; Santos et al., 2020). In the existing prognostic risk assessment models for patients with PMF, apart from driver mutations, additional mutations and the karyotype were taken into account. MIPSS70 (mutation-enhanced international prognostic scoring system for transplant age patients) uses mutations and clinical data, and MIPSS*v2* (the karyotype-enhanced MIPSS70) uses mutations, karyotype, and clinical data; GIPSS (the genetically-inspired prognostic scoring system) is based solely on mutations and karyotype. The use of the NGS technique to assess the risk of patients with PMF is very helpful, especially in a model that is based on genetic changes [(Guglielmelli et al., 2018; Tefferi et al., 2018b; Tefferi et al., 2018c)].

### 3.3 TN-MPN

Recent studies have demonstrated the utility of NGS in refining the characterization of TN-MPNs by establishing clonality and detecting different mutations in driver mutations. Information from sequencing data analysis aids in clinical decision-making. The implementation of the NGS technique helped redefine the diagnosis of TN-MPN. Some studies highlighted the problem of the existence of non-canonical mutations that are not detected by routine methods. Studies have shown the need to search for a clonal marker to confirm the diagnosis. Another problem is that the routine techniques that we use for establishing a diagnosis of MPN have limited sensitivity. NGS helps improve sensitivity, and with this technique, we can even discover mutations in genes that have not been previously associated with MPN (Costa Melo Svidnicki et al., 2019; Geay et al., 2020; Michail et al., 2021; Maddali et al., 2022; Wu et al., 2022).

## 4 Conclusion

In the age of molecular medicine, new advanced technologies are needed to improve laboratory diagnostics. Detection of the driver mutation was a breakthrough discovery in the diagnosis of myeloproliferative neoplasms. This helped determine the pathogenesis of the disease. The introduction of the NGS technique has significantly changed the perception and approach to diagnosis, risk assessment, and treatment of MPN. Thanks to the ability to search for mutations in many genes simultaneously, this technique allows for a more accurate diagnosis based on the patient’s genetic profile. NGS enables the identification of a group of patients with a poor prognosis, patients with high genetic instability, and an increased risk of disease progression or transformation into AML. The identification of high-risk mutations (HMR) and the possibility of incorporating them into prognostic scoring systems highlight the importance of genetic testing in hematooncology.
